# Key Role for Store-Operated Ca^2+^ Channels in Activating Gene Expression in Human Airway Bronchial Epithelial Cells

**DOI:** 10.1371/journal.pone.0105586

**Published:** 2014-08-26

**Authors:** Krishna Samanta, Daniel Bakowski, Anant B. Parekh

**Affiliations:** Department of Physiology, Anatomy and Genetics, University of Oxford, Oxford, United Kingdom; University of Debrecen, Hungary

## Abstract

Ca^2+^ entry into airway epithelia is important for activation of the NFAT family of transcription factors and expression of genes including epidermal growth factor that help orchestrate local inflammatory responses. However, the identity of epithelial Ca^2+^ channel that activates these transcriptional responses is unclear. In many other non-excitable cells, store-operated Ca^2+^ entry is a major route for Ca^2+^ influx and is mediated by STIM1 and Orai1 proteins. This study was performed to determine if store-operated Ca^2+^ channels were expressed in human bronchial epithelial cells and, if so, whether they coupled Ca^2+^ entry to gene expression. Cytoplasmic Ca^2+^ measurements, patch clamp recordings, RNAi knockdown and functional assays were used to identify and then investigate the role of these Ca^2+^ channels in activating the NFAT and c-fos pathways and EGF expression. STIM1 and Orai1 mRNA transcripts as well as proteins were robustly in epithelial cells and formed functional Ca^2+^ channels. Ca^2+^ entry through the channels activated expression of c-fos and EGF as well as an NFAT-dependent reporter gene. Store-operated Ca^2+^ entry was also important for epithelial cell migration in a scrape wound assay. These findings indicate that store-operated Ca^2+^ channels play an important role in stimulating airway epithelial cell gene expression and therefore comprise a novel potential therapeutic target for the treatment of chronic asthma and related airway disorders.

## Introduction

A common theme in chronic asthma is significant remodelling of the airway wall [1]. Changes include an increase in both smooth muscle mass and sensitivity to contractile triggers, accumulation of extracellular matrix below the epithelial basement membrane, appearance of gaps between epithelia and an increase in the number of mucus-producing goblet cells within the epithelial cell layer [2].

Airway epithelia lie at the interface between a host and its environment and thereby comprise a first line of defence against air-borne allergens. Although long considered a passive component to the remodelling process, recent work has now established that airway epithelia respond directly to environmental risk factors associated with asthma [3] and help trigger and then sustain the subsequent allergic cascade [4]. Following allergen-induced activation of cell-surface receptors, airway epithelial cells release a variety of signals that stimulate lung antigen-presenting dendritic cells and attract dendritic cell precursors and other monocytes as well as Th2 lymphocytes [2]. Stimulants released from airway epithelia include ATP, uric acid, lysophosphatidic acid, GM-CSF, CCL2/CCL20 chemokine ligands and a variety of interleukins such as members of the interleukin-1 family [5], [6]. Airway epithelia also release growth factors including epidermal growth factor (EGF) and the closely related amphiregulin and heparin-binding epidermal growth factor-like growth factor, which regulate the remodelling process through activation of the epidermal growth factor receptor [7], [8].

The house dust mite allergen and physiological triggers including histamine increase cytoplasmic Ca^2+^ in airway epithelial cells [9], [10]. Ca^2+^ entry is particularly important for airway epithelial cell function. Ca^2+^ influx is required for EGF secretion [11], [12] and epithelial barrier dysfunction and CCL20 production in response to allergens is dependent on Ca^2+^ entry [9]. In non-excitable cells, a major route for Ca^2+^ influx is through store-operated Ca^2+^ release-activated Ca^2+^ (CRAC) channels in the plasma membrane [13], [14]. These channels activate following the emptying of intracellular Ca^2+^ stores, as occurs following stimulation of G protein-coupled receptors or growth factor receptors that couple to phospholipase C to generate the second messenger inositol 1,4,5-trisphosphate (InsP_3_). The two key components of the CRAC channel pathway are the ER resident protein STIM1, which senses the amount of Ca^2+^ within the store [15], [16], and the pore-forming subunit of the CRAC channels Orai1 [17], [18], [19], [20]. In mast cells and T lymphocytes, Ca^2+^ entry through Orai1 activates the Ca^2+^-dependent transcription factor NFAT [21], [22], [23], [24], which regulates expression of genes encoding chemokines and cytokines. In the immortalised cystic fibrosis bronchial airway epithelial cell line CFBE41o-, transduced with wildtype cystic fibrosis transmembrane regulator, store-operated Ca^2+^ influx was present and required Orai1 expression [25]. Ca^2+^ influx through this pathway increased interleukin 8 expression. Despite its importance in airway epithelial cell remodelling, the molecular identity of the Ca^2+^ influx pathway that activates expression of EGF and other signalling molecules is not clear. Here, we show that store-operated CRAC channels are present and functional in human airway epithelial cells. Ca^2+^ entry through these channels stimulates gene expression including transcription of EGF. We also show that the channels are regulated by cold, a common pre-disposing factor in asthma [26], [27], and are important for epithelial cell migration. CRAC channels are therefore an attractive new therapeutic target for managing airway remodelling.

## Results

### Store-operated Ca^2+^ influx is present in 16HBE cells

**Figure 1:**
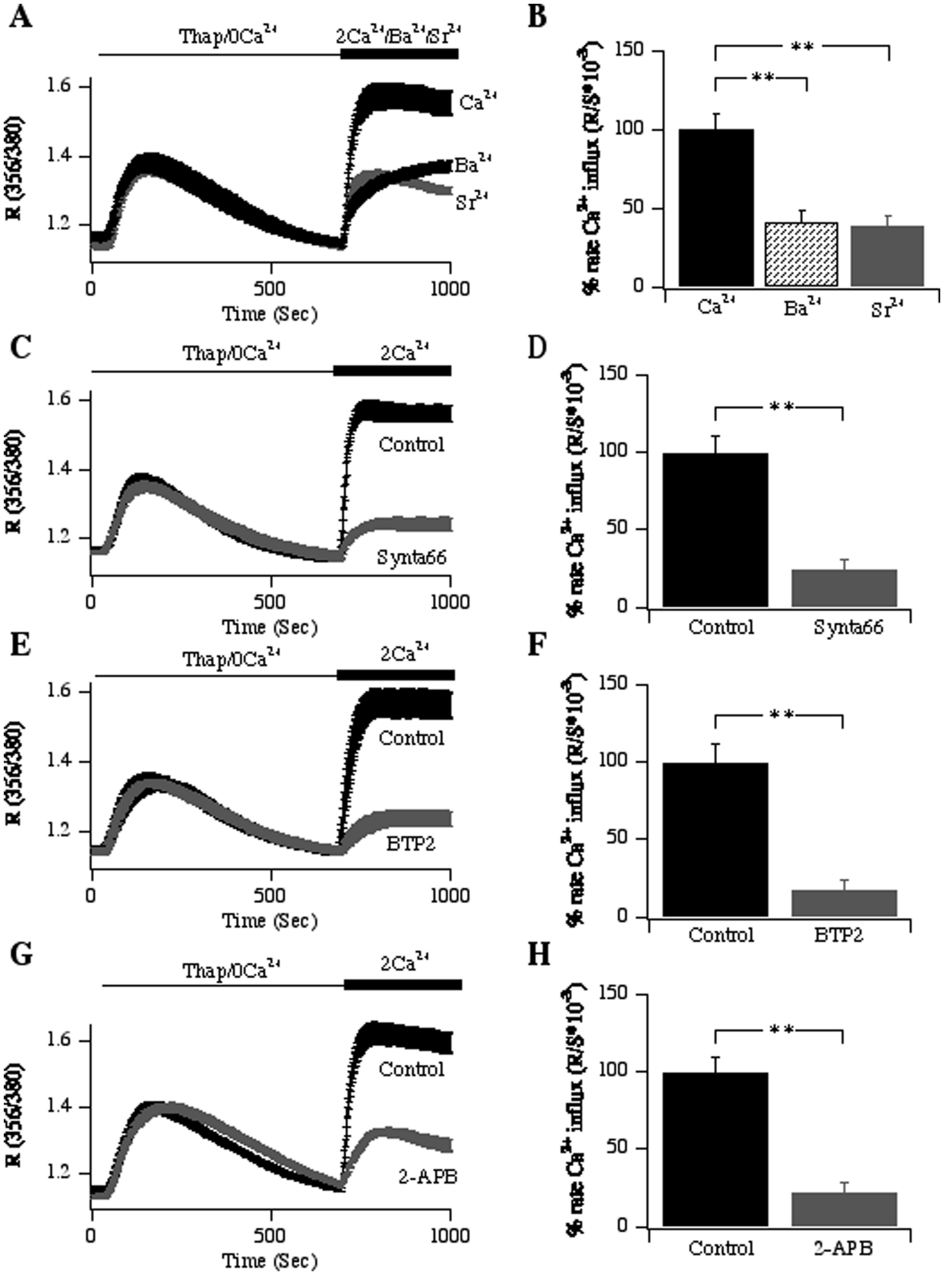
Store-operated Ca^2+^ influx in lung epithelia. A, Following store depletion with thapsigargin (2 µM) in Ca^2+^-free solution, readmission of 2 mM external Ca^2+^ resulted in Ca^2+^ influx. Ba^2+^ and Sr^2+^ were permeable too. B, Aggregate data (normalised to the rate of Ca^2+^ influx) is summarised. Each bar represents between 40 and 55 cells. C-H, Store-operated Ca^2+^ influx is inhibited by the CRAC channel blockers Synta66 (C and D; aggregate data shown represent 53 control cells and 44 cells in the Synta66 group); BTP2 (E and F; aggregate data shown represent 44 control cells and 36 cells in the BTP2 group); 2-APB (G and H; aggregate data shown represent 41 control cells and 59 cells in the 2-APB group). Inhibitors were applied 5 minutes before stimulation with thapsigargin.

We tested for the presence of store-operated Ca^2+^ entry in the human bronchial epithelial cell line (16HBE) by stimulating cells with the sarcoplasmic/endoplasmic reticulum Ca^2+^ATPase inhibitor thapsigargin (2 µM) in Ca^2+^-free external solution [28], [29]. By blocking Ca^2+^ uptake into the stores, thapsigargin unmasks a Ca^2+^ leakage pathway that gradually leads to Ca^2+^ store depletion. Once Ca^2+^ release to thapsigargin had terminated, we readmitted Ca^2+^ to the external solution. A rapid rise in cytoplasmic Ca^2+^ occurred, indicating the presence of store-operated influx (Figure 1A). We quantified this by differentiating the Ca^2+^ response arising from Ca^2+^ influx (Figure 1B), as the rate of rise is a better indicator of channel activity than the steady-state Ca^2+^ signal. CRAC channels also conduct Ba^2+^ and Sr^2+^ [30], [31]. Both divalent cation permeabilities increased after thapsigargin stimulation (Figure 1 A, B), consistent with the presence of functional CRAC channels.

### Pharmacological profile of store-operated Ca^2+^ influx

**Figure 2:**
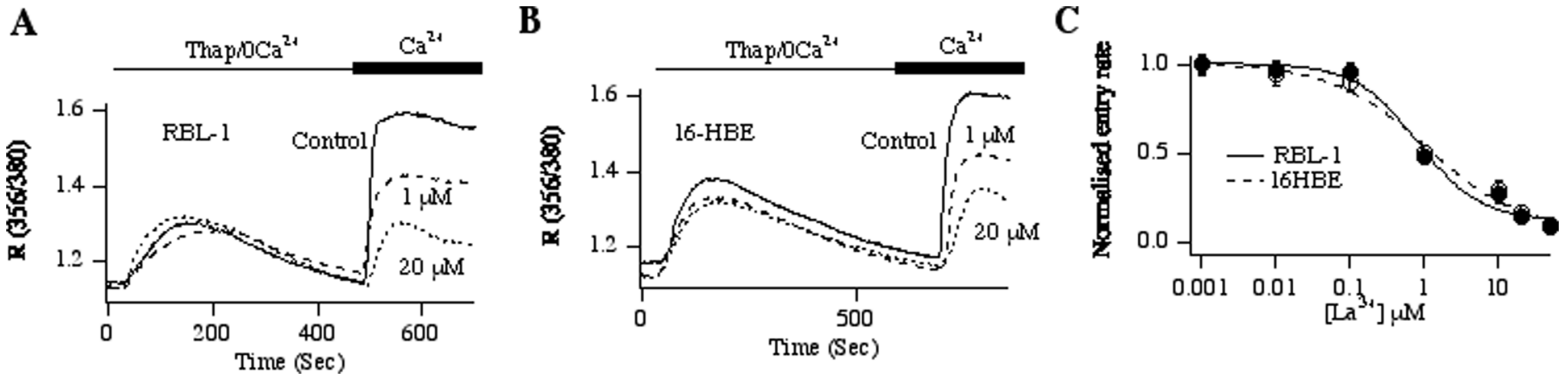
La^3+^ blocks store-operated Ca^2+^ entry in 16HBE cells. A, Ca^2+^ influx is inhibited in a dose-dependent manner by La^3+^ in RBL-1 cells. B, The inhibition of Ca^2+^ influx by La^3+^ in epithelial cells is shown, taken from identical conditions to that in panel A and used on the same days. C, Dose-inhibition curves for the two cell types are compared. Data are fitted with a Hill-type equation of the form: % Inhibition  = [La^3+^]^n^/([La^3+^]^n^+X^n^) where n is the Hill coefficient and X is the IC_50_. The Y-axis denotes the normalised rate of Ca^2+^ entry, in the presence of different concentrations of La^3+^. A value of 1.0 represents the entry rate in the absence of La^3+^.

CRAC channels in immune cells are blocked by a range of molecules including Synta66 [32], BTP2 [33] and 2-APB [34]. To see whether CRAC channels in 16HBE cells exhibited a similar profile, we examined the impact of these inhibitors on Ca^2+^ influx evoked by thapsigargin. Synta66 (10 µM; Figure 1C, D), BTP2 (10 µM; Figure 1E, F) and 2-APB (20 µM; Figure 1G, H) all inhibited Ca^2+^ influx without compromising the rate or extent of Ca^2+^ release from the stores. The ability of the trivalent cations Gd^3+^ and La^3+^ to inhibit Ca^2+^ flux through human Orai1 is reduced by mutating negatively charged aspartate residues (D110, D112) in the extracellular loop between transmembrane domains I and II, which are close to the glutamate residue (E106) that confers Ca^2+^ selectivity [19], [35]. Similar concentration-dependent inhibition of Ca^2+^ entry in 16HBE cells and immune cells by La^3+^ would therefore reinforce the view that the Ca^2+^ entry pathway activated by thapsigargin in 16HBE cells is indeed the CRAC channel. We therefore compared the concentration dependence of block of Ca^2+^ influx by La^3+^ between 16HBE and RBL-1 cells, the latter being a model CRAC channel system. La^3+^ inhibited store-operated Ca^2+^ influx in RBL-1 cells in a dose-dependent manner (Figure 2A), and the relationship could be fitted with a Hill-type equation that yielded an IC_50_ of 0.8 µM and Hill coefficient of 1.0. In 16HBE cells, La^3+^ also inhibited Ca^2+^ influx in a concentration-dependent manner (Figure 2B), revealing an IC_50_ 1.1 µM of and Hill coefficient of 1.0 (Figure 2C), values that were similar to those seen in RBL-1 cells. Collectively, the pharmacological profile of store-operated Ca^2+^ entry in 16HBE cells would be consistent with the presence of CRAC channels.

### Patch clamp recordings reveal a Ca^2+^-selective inwardly rectifying current activated by store depletion

**Figure 3:**
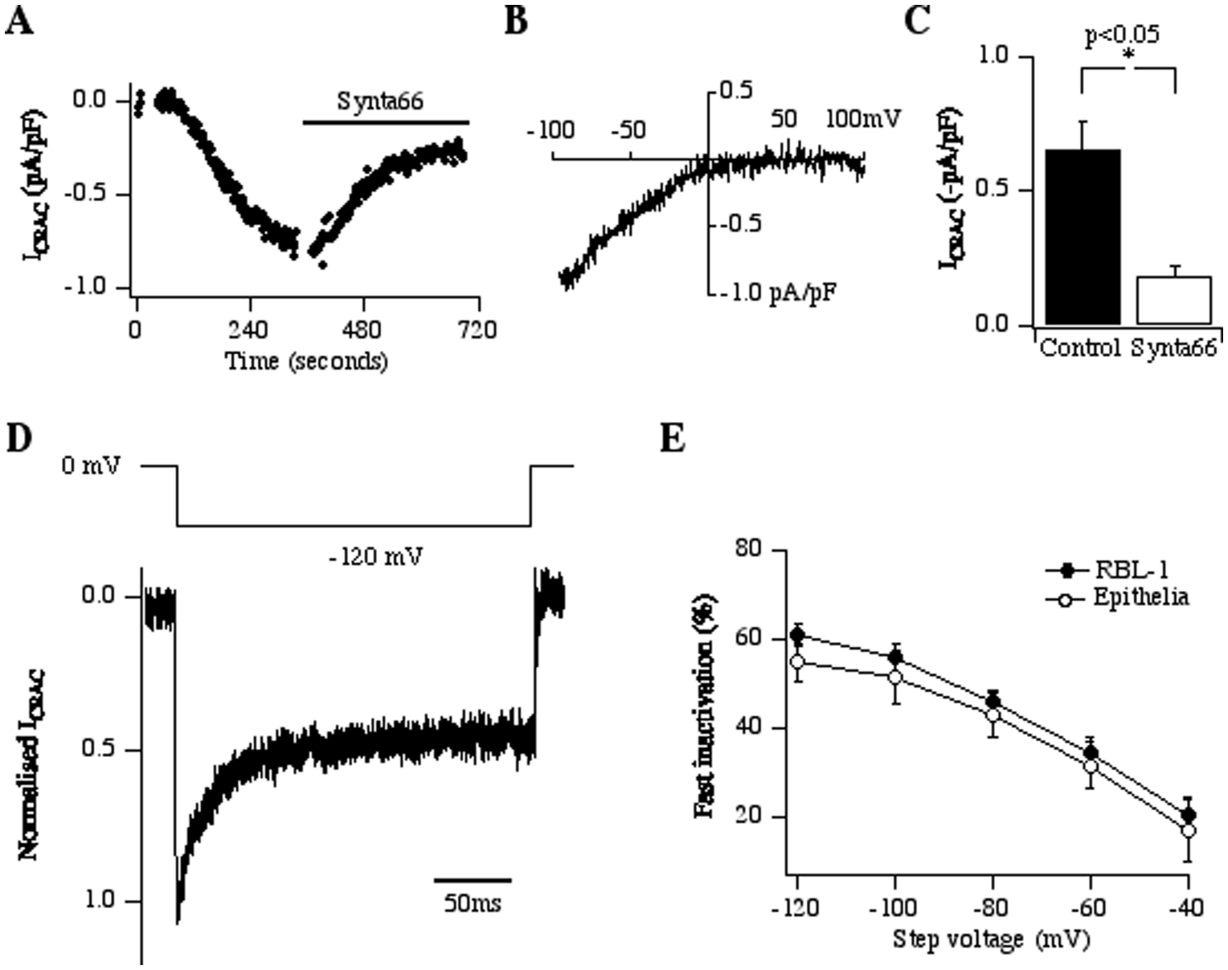
Whole cell patch clamp recordings demonstrate the presence of CRAC channels. A, Passive store depletion by inclusion of 10 mM EGTA in the pipette activates a slowly developing inward current that is inhibited by 10 µM Synta66. B, Current-voltage-relationship taken at 270 seconds from panel A. C, Aggregate data are compared. Current was measured at −80 mV. Control bar is from 7 cells and Synta66 group is from 5 cells. D, Fast inactivation of the CRAC current in epithelia is shown. Upper panel depicts the voltage protocol and lower panel shows the current during the pulse to −120 mV. Pipette solution was the same as in panel A. E, Aggregate data is compared between RBL-1 and 16HBE cells. Holding potential was 0 mV. Each point is the average of between 3 and 6 cells.

We carried out whole cell patch clamp experiments to test directly for the presence of functional CRAC channels. Dialysis with a pipette solution containing 10 mM EGTA to deplete the stores passively resulted, after a delay of ∼50–100 seconds, in the development of an inward current that activated slowly (Figure 3A). The current-voltage relationship revealed a non-voltage-activated, inwardly rectifying current with a reversal potential >+30 mV (Figure 3B), similar to the well-characterised CRAC current seen in mast cells [13], [36]. Dialysis with InsP_3_ (30 µM) and 10 mM EGTA activated a Ca^2+^ current with an identical current-voltage relationship, but the current activated more quickly (half-time of 32 seconds). The mean size of the current (measured at −80 mV) was ∼-0.8 pA/pF (Figure 3C), approximately 3 times smaller than the size of CRAC current in RBL-1 cells following passive store depletion [37]. The current was blocked by Synta66 (Figure 3A and 3C) and did not develop in the absence of external Ca^2+^. A hallmark of CRAC channels is Ca^2+^-dependent fast inactivation, which develops within tens of milliseconds and arises through a negative feedback mechanism triggered by permeating Ca^2+^ ions acting within a few nm of the intracellular mouth of the channel [13], [38]. Fast inactivation increases with hyperpolarization as the driving force for Ca^2+^ entry increases. We have previously characterized fast inactivation of CRAC channels in detail in RBL-1 cells [31]. A hyperpolarization to −120 mV initially increased the CRAC current in epithelial cells but then the amplitude declined over milliseconds due to fast inactivation (Figure 3D). Using identical recording conditions, we found that the extent of fast inactivation over a broad voltage range was indistinguishable between RBL-1 and 16HBE cells (Figure 3E), consistent with the presence of CRAC channels in the epithelial cell line.

### STIM and Orai are present and functional in 16HBE cells

**Figure 4:**
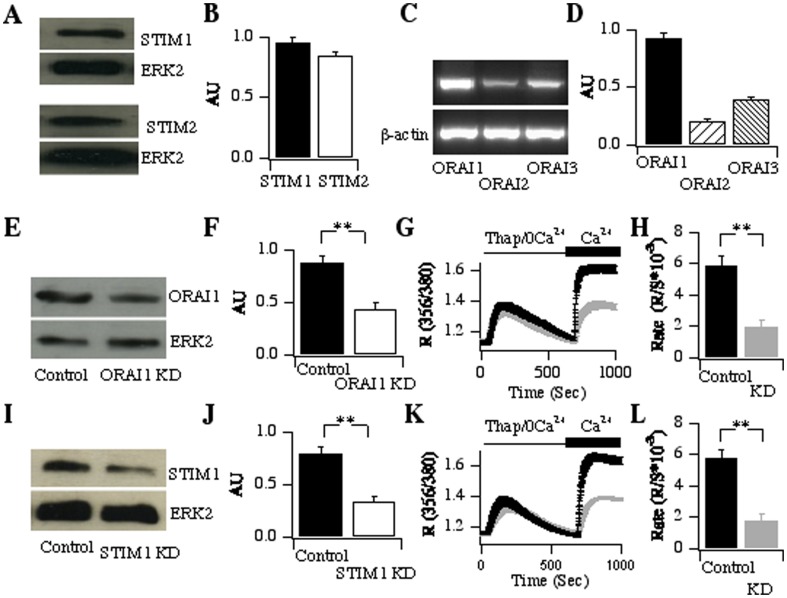
STIM1 and Orai1 are expressed and functional in 16HBE cells. A–B, Western blots show the presence of STIM1 (A) and STIM2 (B) in 16HBE lysates. Aggregate data from 3 independent experiments are shown in B. C, RT-PCR reveals the presence of Orai transcripts. D, Averaged data are compared. E, Western blot shows the presence of Orai1 protein and that siRNA construct to knock down Orai1 (labelled Orai1 KD) reduces protein expression. F, The histogram summarises the extent of Orai1 knockdown from 3 independent experiments. G, Ca^2+^ measurements show that knock down of Orai1 reduces the rate and extent of Ca^2+^ influx. H, Aggregate data are summarised. Control group is the average of 63 cells and Orai1 KD group is 81 cells. I, Western blot shows that siRNA against STIM1 reduces protein expression. J, Aggregate data from 4 independent experiments are compared. K, Store-operated Ca^2+^ influx is reduced after knockdown of STIM1. L, Histogram compares the rate of Ca^2+^ influx for the two conditions. Control group is 45 cells, STIM1 KD group is 59 cells.

The CRAC channel is comprised of the ER Ca^2+^ sensor STIM1 and the plasma membrane pore-forming subunit Orai1. Western blots revealed the presence of both STIM1 (Figure 4A; upper panel) and STIM2 protein (Figure 4A; lower panel) in 16HBE cells. Densitometric analysis on several independent gels suggested that the proteins were present at similar levels (Figure 4B). RT-PCR experiments demonstrated that Orai1 was also present (Figure 4C), along with Orai2 and Orai3, although the latter two were at considerably lower levels (Figure 4D). Western blotting confirmed the presence of Orai1 protein (Figure 4E). To see whether the expressed proteins were functional, we used siRNA approaches to reduce expression and then observed the impact on store-operated Ca^2+^ influx. We achieved ∼55% knockdown of Orai1 protein (Figure 4 E, F) and this resulted in ∼70% reduction in Ca^2+^ influx to thapsigargin (Figure 4G, H). Knockdown of STIM1 reduced protein expression by ∼60% (Figure 4 I, J), and this was associated with a reduction in store-operated Ca^2+^ influx of ∼70% (Figure 4 K, L).

### CRAC channels activate c-fos and NFAT-dependent gene transcription

**Figure 5:**
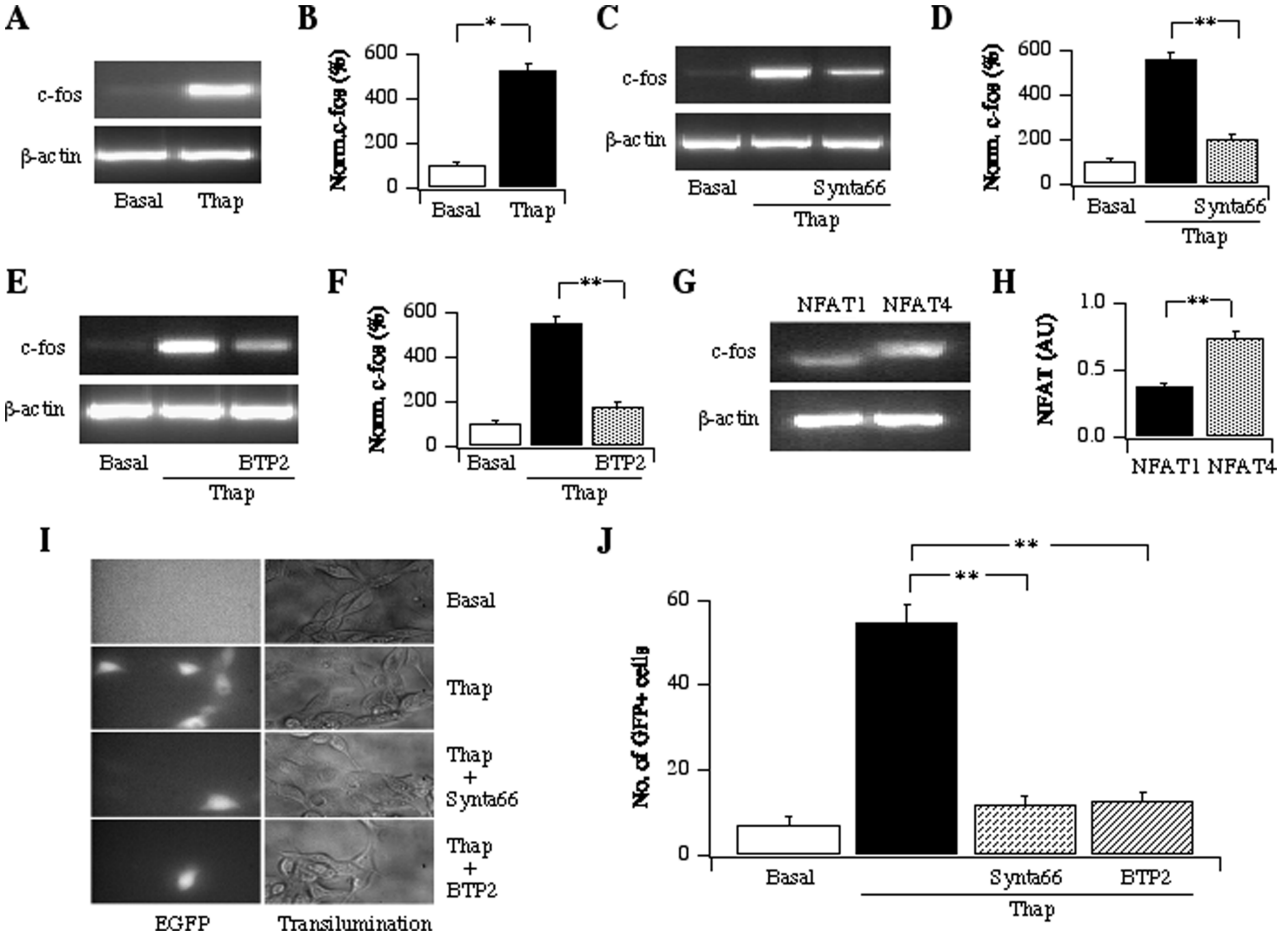
CRAC channels activate gene expression in 16HBE cells. A, C-fos mRNA is increased by thapsigargin. B, Aggregate data from 4 experiments are summarised. C-F, c-fos expression induced by thapsigargin is reduced by Synta66 (C and D) and BTP2 (E and F). G, RT-PCR reveals the presence of mRNA for NFAT1 and NFAT4. H, Relative levels of NFAT are compared. I, NFAT-dependent GFP reporter gene expression is compared for the different conditions. J, Aggregate data from 3 independent experiments are compared. Basal denotes untreated cells.

Epithelial cell remodelling requires gene expression [1]. To see whether Ca^2+^ entry through CRAC channels could impact on nuclear gene expression, we stimulated 16HBE cells with thapsigargin and measured expression of c-fos, a transcription factor that is involved in the initial stages of a local inflammatory response. Relatively brief stimulation (5 minutes) was sufficient to induce robust c-fos transcription 40 minutes later (Figure 5A, B). Transcription of c-fos was significantly reduced by pre-treatment with the CRAC channel blockers Synta66 (Figure 5 C, D) or BTP2 (Figure 5E, F). C-fos hetero-dimerises with c-jun to form the AP-1 complex that interacts with the Ca^2+^-dependent NFAT (nuclear factor of activated T cells) family of transcription factors. RT-PCR studies demonstrated the presence of NFAT1 and NFAT4 in 16HBE cells (Figure 5G, H). To monitor endogenous activation of NFAT, we used a reporter gene system in which GFP was under an NFAT promoter [21]. GFP expression was very low in resting cells but increased significantly after stimulation with thapsigargin (Figure 5I). Pre-exposure to either Synta66 or BTP2 prevented NFAT-driven gene expression in response to thapsigargin (Figure 5I, J).

### EGF transcription is increased following CRAC channel activation

**Figure 6:**
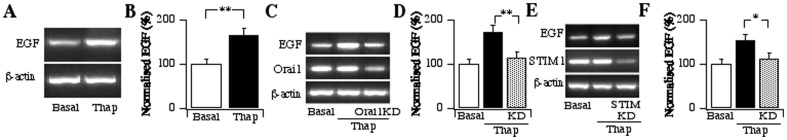
CRAC channels increase EGF expression. A, EGF mRNA levels are increased by thapsigargin. B, Aggregate data from 4 experiments are compared. C, The increase in EGF mRNA after thapsigargin stimulation is reduced following knockdown of Orai1 D, Aggregate data from 3 experiments are compared. KD here denotes knock down of Orai1. E, STIM1 knockdown reduces EGF mRNA levels. Basal denotes untreated. F, Aggregate data from 3 experiments are compared. KD denotes STIM1 knockdown.

EGF is generated in a manner dependent on Ca^2+^ entry [11], [12]. We therefore hypothesized that Ca^2+^ influx through CRAC channels stimulated EGF transcription. Although there was resolvable basal production of EGF in 16HBE cells, stimulation with thapsigargin for 1 hour resulted in a further significant increase in EGF mRNA (Figure 6A and B). Knockdown of Orai1 (Figure 6C-D) reversed the increase in EGF mRNA following stimulation with thapsigargin. siRNA against Orai1 reduced protein expression by ∼60% (Figure 4E) and also reduced Orai1 mRNA by ∼60% (Figure 6C). Similar results were seen following knockdown of STIM1 (Figure 6E–F). Interestingly, basal EGF transcription was not reduced by siRNA targeted knockdown of Orai1, suggesting that either the remaining Orai1 channels were sufficient to maintain basal levels or than an alternative constitutive Ca^2+^ entry pathway was involved.

### Transient cold exposure increases store-operated Ca^2+^ entry

**Figure 7:**
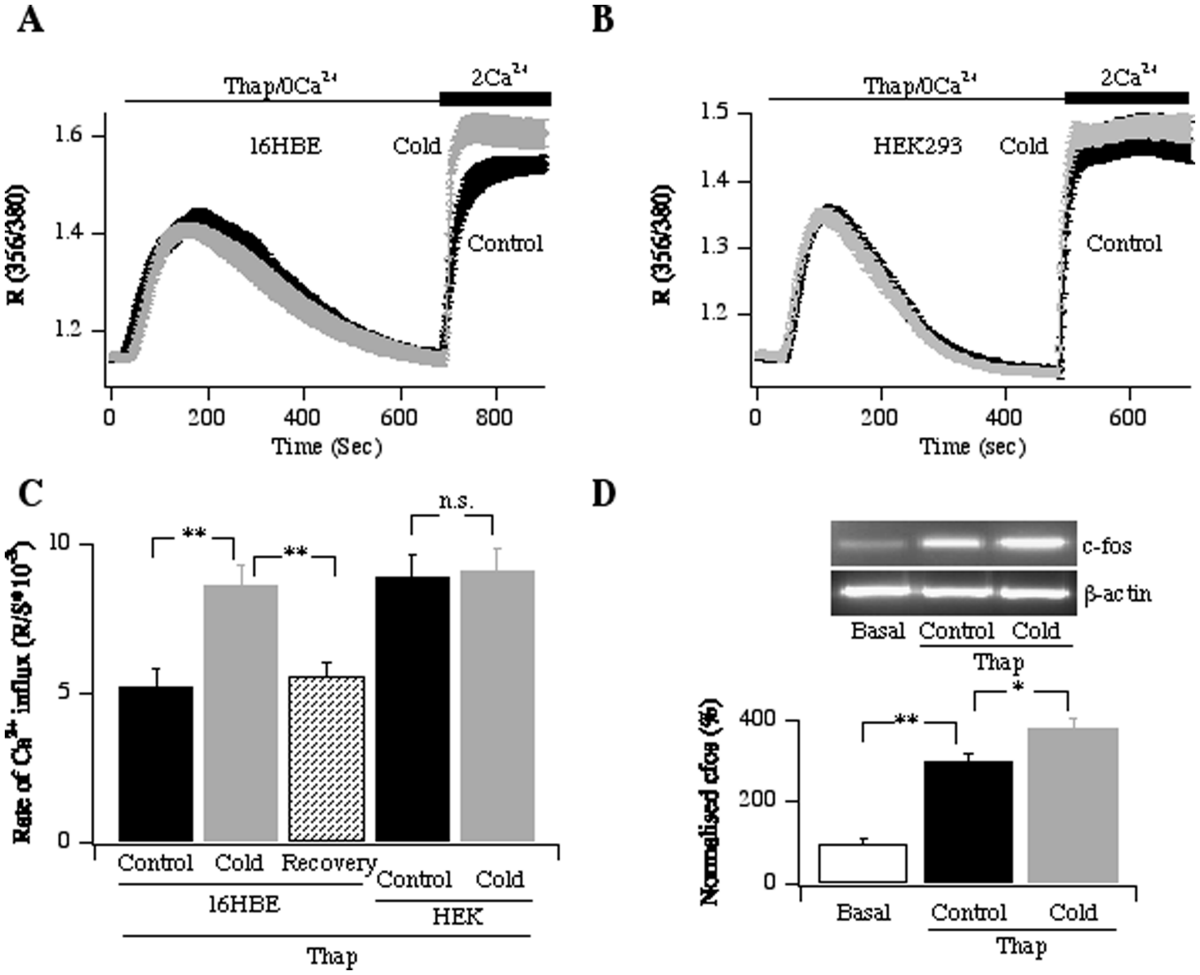
Exposure to cold transiently increases store-operated Ca^2+^ influx in 16HBE cells. A, Ca^2+^ entry was increased in cells pre-exposed to a cold shock. B, Effect of cold shock on store-operated Ca^2+^ entry in HEK293 cells. C, Rate of Ca^2+^ influx is compared between the different conditions. Control denotes cells not exposed to a cold shock. Recovery refers to cells exposed to a cold shock but allowed to recover at 37°C overnight hours. Each bar is the average of >88 cells. D, RT-PCR measurements of cfos mRNA, U denotes untreated. D, Aggregate data from 4 independent experiments compares the increase in c-fos mRNA.

An important predisposing factor to the development of asthma is acute exposure to cold [27]. We introduced a transient cold shock by exposing 16HBE cells to 15°C for 2 hours and then, after incubation at 37°C for 30 minutes, we measured Ca^2+^ entry evoked by thapsigargin. Although the Ca^2+^ release phase was similar between control cells and those exposed to cold, the rate and extent of Ca^2+^ entry was significantly faster in the cold-treated cells (Figure 7 A, C). Increasing the recovery time from cold exposure from 30 minutes to ∼16 hours reversed the stimulatory effects on store-operated Ca^2+^ influx (Figure 7C). We checked to see whether cold exposure affected store-operated Ca^2+^ entry in other cells. Because the rate of Ca^2+^ influx in non-cold-exposed RBL-1 cells after store depletion is very high, we turned to HEK293 cells because they have only a slighter faster rate of influx compared with that seen in 16HBE cells. Cold exposure did not accelerate the rate of Ca^2+^ entry in HEK293 cells (Figure 7B, C).

The accelerated rate of Ca^2+^ influx in 16HBE cells was functionally important because it led to a significant increase in c-fos transcription (Figure 7C). In this experiment, we evoked Ca^2+^ influx for just 60 seconds (by adding back Ca^2+^ for 60 seconds to cells pre-treated with thapsigargin in Ca^2+^-free solution) in order to dissect out more clearly the impact of the modest increase in Ca^2+^ entry rate from the effects of a prolonged cytoplasmic Ca^2+^ rise that occurs when responses are measured after several minutes stimulation.

### CRAC channels regulate epithelial migration

**Figure 8:**
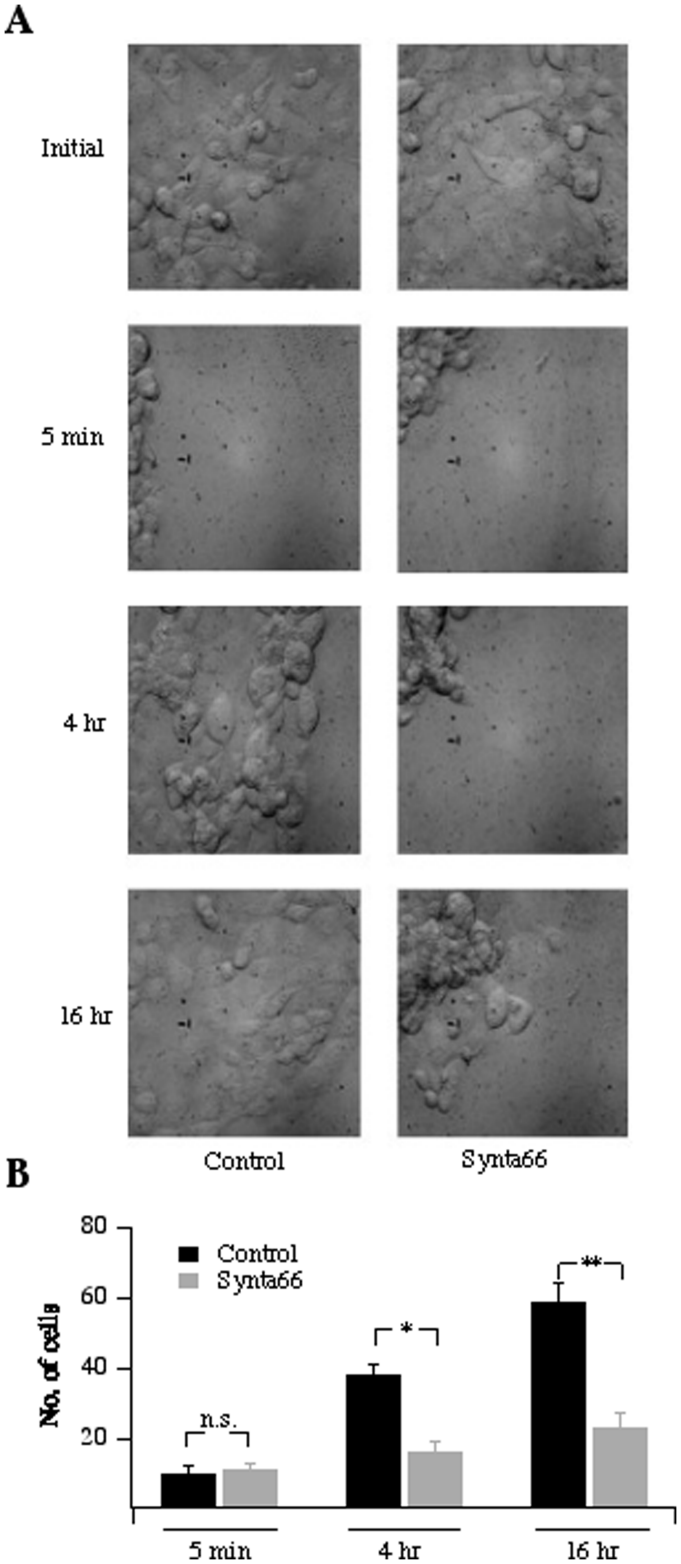
CRAC channel activity is required for 16HBE cell migration in a scrape wound assay. A, transillumination images of cells prior to a scrape wound are shown. After scraping, images are shown at the times indicated for control cells and those continually exposed to Synta66. B, Aggregate data from three independent scrape would assays are compared. Several snapshots were taken per culture dish and the averaged number of cells per image are plotted.

CRAC channels have been shown to contribute to migration of vascular myocytes in a scratch wound migration assay [39]. Following this approach, we introduced a scratch wound and tracked the rate at which 16HBE cells repopulated the spaces within the wound. In control cells, no migration occurred shortly after inducing the wound but repopulation was significant 4 hours later and almost complete after 16 hours (Figure 8A and 8B). By contrast, treatment with Synta66 immediately after the scratch wound substantially slowed the rate at which the cells migrated into the wound area (Figure 8A and B).

## Discussion

Although Ca^2+^ influx stimulates both rapid and long-lasting responses in epithelial cell function, very little is known about the identity of the Ca^2+^ entry pathways present. In this study, we show that store-operated CRAC channels are functionally expressed in airway epithelia where they stimulate EGF gene expression and are required for cell migration. We also find that CRAC channel activity increases following exposure to cold air, an environmental factor that contributes to airway inflammation.

Orai1 was strongly expressed in the airway epithelial cells, both at mRNA transcript and protein levels. Orai3 mRNA was also clearly resolvable, although to a lesser extent than Orai1. Both STIM1 and STIM2 proteins were also expressed and at similar levels. Store-operated Ca^2+^ influx was present in the epithelia and several lines of evidence suggest the underlying channels are largely indistinguishable from the well-characterised CRAC channels in mast cells, T lymphocytes and related cell lines [34]. Firstly, whole cell patch clamp recordings identified a Ca^2+^ current that was activated by store depletion, was non-voltage activated, showed strong inward rectification, was Ca^2+^-selective, exhibited Ca^2+^-dependent fast inactivation that had a similar voltage-dependence to that seen in mast cells, and was inhibited by the CRAC channel blocker Synta66 [40], [41]. Secondly, Ca^2+^ flux measurements identified permeability to Ca^2+^, Sr^2+^ and Ba^2+^, as is the case for CRAC channels [30], [36]. Thirdly, the CRAC channel pore blocker La^3+^ inhibited store-operated Ca^2+^ influx in airway epithelia and RBL-1 mast cells with similar IC_50_ and Hill coefficients. Fourthly, structurally distinct small molecule inhibitors of CRAC channels in immune cells also blocked Ca^2+^ influx in airway epithelia. Finally, siRNA directed against the two key CRAC channels components, STIM1 and Orai1, reduced protein expression and Ca^2+^ influx to similar extents.

We also found that activation of CRAC channels had important consequences on epithelial cell function. Ca^2+^ influx through the channels activated both c-fos and NFAT transcription factors as well as NFAT-driven gene expression. Ca^2+^ entry through CRAC channels also increased transcription of EGF, which plays an important role in epithelial cell repair and regeneration [1]. In a scrape wound assay, epithelial cell migration was reduced considerably following CRAC channel inhibition. Hence functional CRAC channels are involved in the repair programme. EGF has been shown to play an important role in bronchial epithelial repair [42] and it is therefore particularly noteworthy that CRAC channels increase EGF transcription. K^+^ channel blockers have also been found to impair EGF-driven epithelial repair [42] and it is possible that this is a consequence of the reduced Ca^2+^ influx through CRAC channels that arises when the membrane potential is depolarised after K^+^ channel inhibition.

Exposure to cold air is a pre-disposing factor to the development of asthma and other airway diseases. CRAC channel activity increased ∼1.5 fold after transient pre-exposure to cold [26], [27] and this was associated with a small increase in c-fos gene transcription. CRAC channel activity increased after ∼2 hours of cold exposure and this was not impaired by inhibition of protein synthesis with tunicamycin (applied together with the cold shock; data not shown), suggesting that the increase in Ca^2+^ entry did not involve increased protein expression. Heating cells to >35°C leads to clustering of STIM1 proteins at endoplasmic reticulum-plasma membrane junctions and results in transient Ca^2+^ entry through Orai1 upon cooling [43]. A cooling period followed by rewarming transiently increased Ca^2+^ entry in epithelial cells, suggesting reduced temperature might impair an inhibitory pathway. Regardless of the mechanism, our findings reveal that a pre-disposing factor linked to asthma susceptibility increases Ca^2+^ influx and thereby accelerates transcription of c-fos, a regulator of chemokine and cytokine production. CRAC channels might therefore operate in parallel with cold-sensing TRPM8 channels that have been found to trigger mucin hypersecretion in human bronchial epithelia [44].

Collectively, our results identify CRAC channels as an important route for Ca^2+^ entry into airway epithelia that drives expression of genes involved in the remodelling mechanism. Targeting CRAC channels therefore might provide a new therapeutic approach for managing chronic airway inflammatory disorders including asthma and chronic obstructive pulmonary disease.

## Experimental

### Cell culture

The human bronchial airway epithelial cell line 16HBE was a kind gift from Dr Lin-Pei Ho (Weatherall Institute of Molecular Medicine, Oxford). Cells were cultured (37°C, 5% CO_2_) in Dulbecco's modified Eagle's medium with 10% fetal bovine serum, 2 mm l-glutamine, and penicillin/streptomycin.

### Transfection

16HBE cells were transfected using Lipofectamine system (Lipofectamine LTX & Plus Reagent, which were purchased from Invitrogen), using the manufacturer's instructions. Transfection efficiency was ∼60%, judged from the fraction of GFP positive cells obtained 48 h post transfection.

### Cytoplasmic Ca^2+^ measurements

Cells were loaded with Fura-2/AM (1 mM) for 40 minutes at room temperature in the dark and then washed three times with a standard external solution composed of 145 mM NaCl, 2.8 mM KCl, 2 mM CaCl_2_, 2 mM MgCl_2_, 10 mM D-glucose, 10 mM HEPES, pH 7.4 with NaOH, as described [45]. Cells were left for 15 minutes in the dark to allow further de-esterification. Ca^2+^-free solution contained 145 mM NaCl, 2.8 mM KCl, 2 mM MgCl_2_, 10 mM D-glucose, 10 mM HEPES, 0.1 mM EGTA, pH 7.4 with NaOH. Cytoplasmic Ca^2+^ imaging experiments were carried out using a TILL Photonics system with an IMAGO CCD camera. Cells were alternately excited at 356 and 380 nm, and images were acquired every 2 seconds. Images were analyzed off line using IGOR Pro for Windows. Ca^2+^ signals are represented at the 356/380 nm ratio.

### Patch clamp recordings

Whole cell patch clamp recordings were carried out as previously described [36]. Pipettes were pulled from borosilicate glass, were Sylgard-coated and fire-polished. Pipette resistances were in the range 4–6 MΩ when filled with a pipette solution containing (in mM): Cs glutamate 145, NaCl 8, MgCl_2_ 1, Ethylene glycol-bis(b-aminoethyl ether)-N,N,N′,N′,-tetraacetic acid (EGTA) 10, HEPES 10, Mg-ATP 2, pH 7.2 with CsOH. Bath solution contained (in mM): NaCl 135, KCl 2.8, CsCl 10, CaCl_2_ 10, MgCl_2_ 2, HEPES 10, D-glucose 10, pH 7.4 with NaOH. The CRAC current was measured by applying voltage ramps (−100 to +100 mV in 50 msec) at 0.5 Hz from a holding potential of 0 mV. For fast inactivation, step pulses (250 msec duration) were applied from 0 mV to −100 mV every 2 seconds. Currents were filtered using an 8-pole Bessel filter at 2.5 kHz and digitised at 100 ms. Fast inactivation was determined by dividing the steady state current during the hyperpolarising pulse (measured after 240 ms) by the initial current (measured after 1 ms). Capacitative currents were compensated before each ramp by using the automatic compensation of the EPC 9 -2 amplifier. Leak currents were subtracted by averaging 2-3 ramp currents obtained just before I_CRAC_ had started to develop, and then subtracting this from all subsequent currents.

### Western blot

Total cell lysates (50 µg) were separated by SDS-PAGE on a 10% gel and electrophoretically transferred to nitrocellulose membrane, as described^21^. Membranes were blocked with 5% non-fat dry milk in TBS plus 0.1% Tween 20 (TBST) buffer for 1 hour at room temperature. Membranes were washed with TBST three times and then incubated with primary antibody overnight at 4°C. Anti-STIM1 and –STIM2 antibodies were obtained from Cell Signalling Technology and used at 1∶1000 dilutions. Orai1 and ERK2 antibodies were from Santa Cruz Biotechnology and were used at a dilution of 1∶500 and 1∶5000, respectively. The membranes were then washed with TBST again and incubated with a 1∶2500 dilution of goat anti-rabbit secondary antibody IgG from Santa Cruz Biotechnology for 1 h at room temperature. After washing with TBST, the bands were developed for visualization using ECL-plus Western blotting detection system (GE Healthcare). Gels were quantified using the UN-SCAN-IT software package (Silk Scientific).

### RT-PCR

16HBE cells were stimulated with 2 µM thapsigargin for 10 minutes (for c-fos measurements) or 1 hour (for EGF measurements) at room temperature in standard external solution. Thereafter, cells were washed with Ca^2+^-free external solution (containing 0.1 mM EGTA) without thapsigargin. After a further 40 minutes (at room temperature.), total RNA was extracted by using an RNeasy Mini Kit (Qiagen), as described. RNA was quantified spectrophotometrically by absorbance at 260 nm. Total RNA (1 µg) was reverse-transcribed using the iScriptTM cDNA Synthesis Kit (Bio-Rad), according to the manufacturer's instructions. Following cDNA synthesis, PCR amplification was then performed using BIOX-ACTTM. ShortDNAPolymerase (Bioline) with primers specific for the detection of c-fos (sense, 5′-CCAACCTGCTGAAGGAGAAG-3′, and antisense, 5′-ATGATGCTGGGAACAGGAAG-3′), EGF (sense, 5′-AGGGAAGATGACCACCACTATTCC-3′, and antisense, 5′-TTTTCGATAGCAGCTTCTGAGTCC-3′), NFAT1 (sense, 5′-AGAAACTCGGCTCCAGAATCC-3′, and antisense, 5′-TGGTTGCCCTCATGTTGTTTTT-3′), NFAT4 (sense, 5′-ACCAGCCCGGGAGACTTCAATAGA-3′, and antisense, 5′-AAATACCTGCACAATCAATACTGG-3′), ORAI1 (sense, 5′-CTGCTCATCGCCTTCAGTGC-3′, and antisense, 5′-TCCTTGACCGAGTTGAGATTGTG-3′), ORAI2 (sense, 5′-TGCTGAGCTTAACGTGCCTATC-3′, and antisense, 5′-AGGTGACCAGTTCCAGGTAGC-3′), ORAI3 (sense, 5′-GAGCAACATCCACAACCTCAAC-3′, and antisense, 5′-ACCAGGACAACTTCAGCAAGG-3′), STIM1 (sense, 5′-TGGAGCTGGCACAGTATCAG-3′, and antisense, 5′-TGATTGTCCCGAGTCAACAG-3′) and β-actin (sense, 5′-TTGTAACCAACTGGGACGATATG-3′, and antisense, 5′-GATCTTGATCTTCATGGTGCTAGG-3′) were synthesized by Invitrogen. The PCR products were electrophoresed through an agarose gel and visualized by ethidium bromide staining.

### Gene expression assay

24–36 hours following transfection with an EGFP-based reporter plasmid that contained an NFAT promoter (gift from Dr Yuri Usachev, University of Iowa), cells were stimulated with thapsigargin (100 nM) and the number of cells expressing EGFP measured subsequently (∼24 hours later), as described^22^. Gene expression was defined as fluorescence 3xSD> cell autofluorescence, measured in non-transfected cells. Cells were stimulated in culture medium and maintained in the incubator for ∼24 hours prior to detection of EGFP.

### Cold shock

16HBE cells plated on coverslips were removed from the incubator (37°C) and kept at 15°C for 2 hours. They were again returned to the incubator for either 30 min or overnight before loading with Fura 2-AM for Ca^2+^ imaging experiments. For RT-PCR experiments, the cells were exposed to 2 µM thapsigargin in Ca^2+^-free external solution for 7 minutes and then 2 mM Ca^2+^ was readmitted for 1 minute. Thereafter, cells were washed with Ca^2+^-free external solution without thapsigargin. After a further 40 minutes (at room temperature), total RNA was extracted as described above.

### Scrape wound assay

16HBE cells were allowed to form a monolayer for 48 h. A 100-µl pipette tip was then used to scrape across the culture dish, which was then washed three times with standard culture medium and then returned to the incubator. Bright-field images were captured 5 minutes, 4 hours and 16 hours later, using a Nikon microscope at x40. The images were analyzed using ImageJ and the total number of cells inside the wound were compared with experiments in which Synta66 (10 µM) had been added immediately after wound formation. The perimeter of the wound was marked on the outward facing base of the dish for identification purposes.

### Statistics

Results are presented as mean±sem. Data were compared using Student's t test or by analysis of variance (ANOVA) for multiple groups. Differences were considered statistically significant at values of p<0.05.
